# Left Atrial Venoarterial Extracorporeal Membrane Oxygenation for Acute Aortic Regurgitation and Cardiogenic Shock

**DOI:** 10.1016/j.jaccas.2021.12.030

**Published:** 2022-03-02

**Authors:** Michael Chiang, Pedro E. Gonzalez, Brian P. O’Neill, James Lee, Tiberio Frisoli, Dee Dee Wang, William W. O’Neill, Pedro A. Villablanca

**Affiliations:** Center for Structural Heart Disease, Henry Ford Hospital, Detroit, Michigan, USA

**Keywords:** aortic regurgitation, cardiogenic shock, LAVA-ECMO, VA-ECMO, AI, aortic insufficiency, LA, left atrial, LAVA-ECMO, left atrial venoarterial extracorporeal membrane oxygenation, LV, left ventricle, MCS, mechanical circulatory support, RA, right atrial, TAVR, transcatheter aortic valve replacement, VA-ECMO, venoarterial extracorporeal membrane oxygenation

## Abstract

A 51-year-old man with past medical history of bioprosthetic aortic valve replacement presented in cardiogenic shock secondary to acute bioprosthesis degeneration with severe aortic regurgitation. Venoarterial extracorporeal membrane oxygenation is contraindicated in patients with severe AI. Use of left atrial venoarterial extracorporeal membrane oxygenation resulted in hemodynamic improvement, allowing patient stabilization for emergency valve-in-valve transcatheter aortic valve replacement. (**Level of Difficulty: Advanced.**)

## Central Illustration

**Figure:**
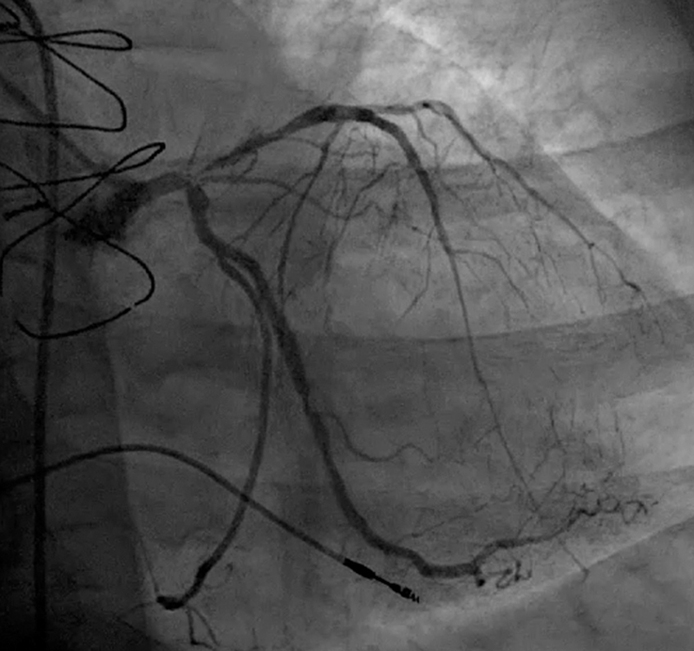

## Introduction

Venoarterial extracorporeal membrane oxygenation (VA-ECMO) has been globally implemented as an emergency means for providing biventricular hemodynamic support in patients with cardiogenic shock. VA-ECMO, however, increases left ventricular (LV) afterload and is contraindicated in patients with severe aortic regurgitation. Left atrial (LA) venoarterial extracorporeal membrane oxygenation (LAVA-ECMO) indirectly unloads the LV by placement of an inflow cannula in the LA and can be considered in such patients.

**Learning Objectives**

• To understand the limitations and contraindications of various MCS devices.
• To understand the technical aspects of LAVA-ECMO insertion.
• LAVA-ECMO is an effective option for patients with cardiogenic shock and severe aortic regurgitation.

## History of Presentation

A 51-year-old man presented to an outside institution with acute chest pain and shortness of breath at rest. His systemic blood pressure was 80/30 mm Hg, and on physical examination, he was found to have a grade 4/6 diastolic and grade 2/6 systolic murmur at the left upper sternal border.

## Past Medical History

The patient was known to have a history of infective endocarditis requiring surgical aortic valve replacement with a 23-mm Freestyle bioprosthesis (Medtronic) with concomitant aortic root repair more than 10 years before presentation. He also had a past medical history of end-stage renal failure on hemodialysis, atrial fibrillation, permanent pacemaker implantation, diabetes mellitus, hypertension, and obstructive sleep apnea.

## Differential Diagnosis

The differential diagnosis included non–ST-segment elevation myocardial infarction, severe aortic regurgitation secondary to degenerative aortic bioprosthesis, severe aortic stenosis secondary to degenerative aortic bioprosthesis, congestive heart failure, and cardiogenic shock.

## Investigations

The electrocardiogram showed no ST-segment elevation. His laboratory profile was remarkable for an elevated high-sensitivity troponin I of 5.6 ng/dL, lactate of 2.6 mmol/L, and B-type natriuretic peptide of 1,248 pg/mL. Bedside surface echocardiogram demonstrated a severely depressed LV ejection fraction of 20% and structural valve degeneration of the bioprosthetic with notable mixed aortic bioprosthetic valve disease, severe aortic regurgitation, and concomitant stenosis.

## Management

**Figure 1:**
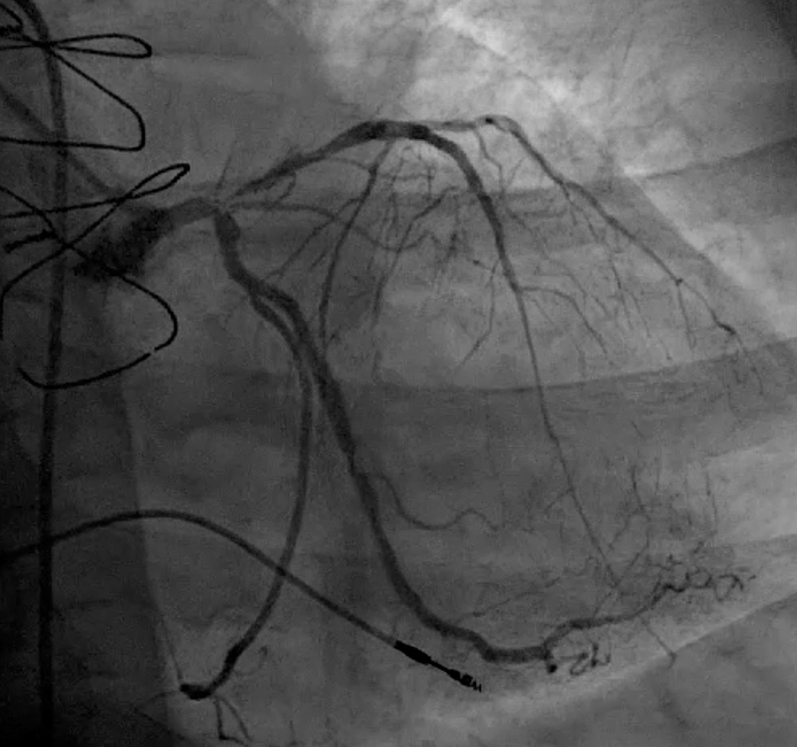
Left Coronary Artery Angiogram

A left heart catheterization was performed, demonstrating severe left main bifurcation coronary artery disease (Figure 1), right coronary artery chronic total occlusion, and an LV end-diastolic pressure of 35 mm Hg. Right heart catheterization was also performed, revealing severely elevated right heart filling pressures (right atrial [RA]: 27 mm Hg) and left heart filling pressures (pulmonary capillary wedge pressure: 44 mm Hg), cardiac index of 0.96 L/min/m^2^, pulmonary artery pulsatility index of 1.2, and aortic valve area 0.53 m^2^ (peak-to-peak gradient: 25 mm Hg). With biventricular failure, in the absence of other means of mechanical circulatory support, an intra-aortic balloon pump was inserted as a bridge to emergency transfer to our institution for escalation of care.

**Figure 2:**
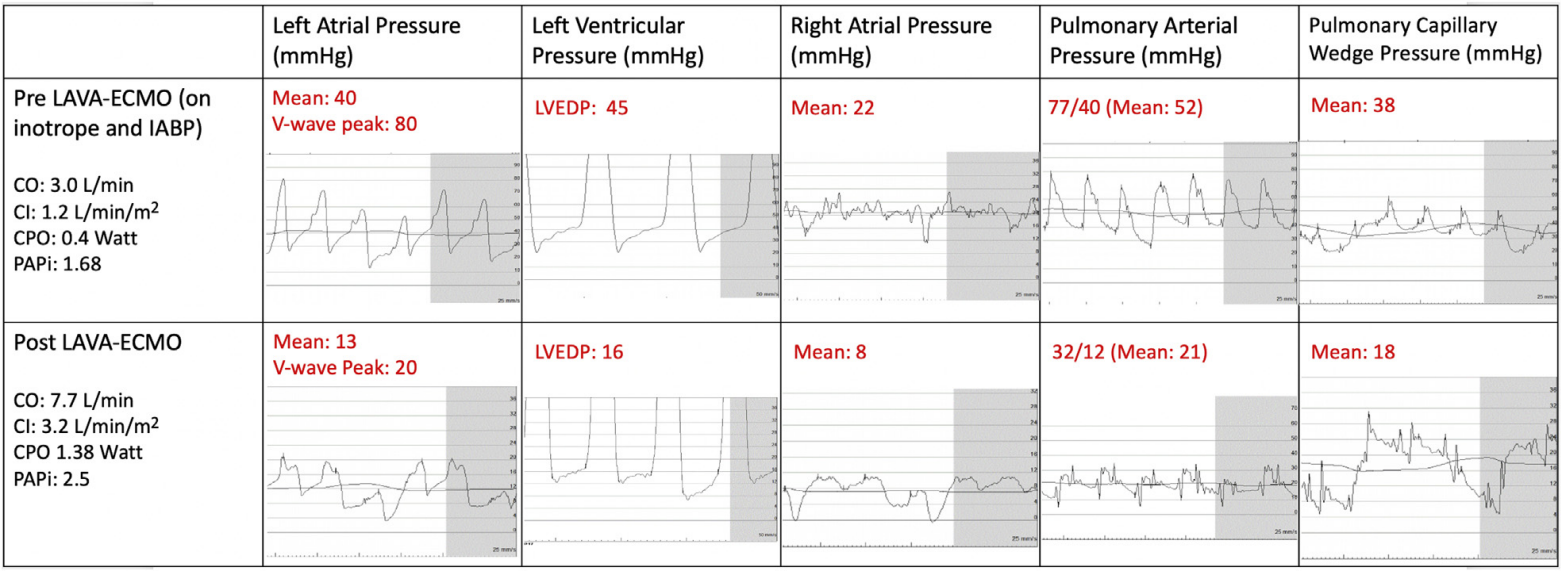
Hemodynamics Before and After LAVA-ECMO CI = cardiac index; CO = cardiac output; CPO = cardiac power output; IABP = intra-aortic balloon pump; LAVA-ECMO = left atrial venoarterial extracorporeal membrane oxygenation; PAPi = pulmonary artery pulsatility index.

**Figure 3:**
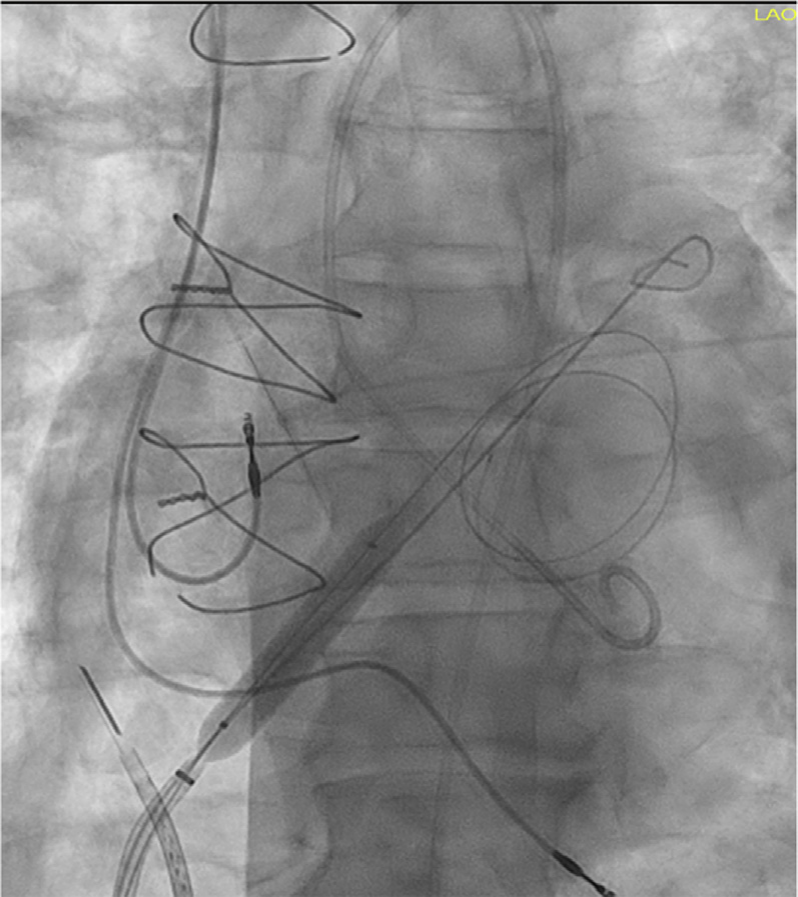
Septostomy With Peripheral Balloon Before Left Atrial Venoarterial Extracorporeal Membrane Oxygenation Venous Cannula Insertion

Upon arrival to the cardiac catheterization laboratory, repeat left and right heart catheterization demonstrated biventricular heart failure with elevated filling pressures (Figure 2). VA-ECMO, which is often used for biventricular support, is contraindicated in this patient with severe aortic insufficiency (AI) because of catastrophic LV overloading. Hence, LAVA-ECMO was implemented. LAVA-ECMO was performed with deep sedation without general anesthesia to avoid risk of further hemodynamic compromise and for dynamic assessment of neurologic function. The arterial ECMO cannula was inserted in a standard fashion. Bilateral femoral venous accesses were obtained. Intracardiac echocardiography catheter 5 was inserted into the RA via venous access. Intracardiac echocardiography–guided transseptal puncture was done via contralateral venous access. A 0.35-mm Amplatz ExtraStiff wire was sent to the left upper pulmonary vein. An 8- × 40-mm peripheral balloon was delivered via the stiff wire for septostomy (Figure 3). A 24-F multifenestrated LAVA-ECMO cannula was then sent across the interatrial septum into the LA. Biatrial and biventricular unloading were feasible, with side holes opening on both the LA and RA sides (Videos 1 and 2). Invasive hemodynamics 30 minutes post–LAVA-ECMO procedure demonstrated an acute decrease in LV end-diastolic pressure by 16 mm Hg from the time of presentation, an RA pressure drop to 8 mm Hg, and an increase in cardiac index to 3.2 L/min/m^2^ (Figure 2).

The patient was evaluated by the cardiothoracic surgeons team and deemed not a surgical candidate for redo aortic valve replacement, given his acute cardiogenic shock presentation with concurrent multiple comorbidities. The patient’s clinical condition stabilized over the following 24 hours with improvement in laboratory markers. The patient was evaluated by the multidisciplinary structural heart team and was recommended for percutaneous coronary intervention of the left main and left anterior descending arteries on day 3 after admission (Video 3A). Valve-in-valve transcatheter aortic valve replacement (TAVR) with a 23-mm Sapien 3 Ultra (Edwards Lifesciences) was performed on day 5 (Video 3B). Hemodynamic assessment after valve-in-valve TAVR valve deployment showed no significant perivalvular leak, and the patient was able to undergo LAVA-ECMO decannulation on the table. Repeat transthoracic echocardiogram demonstrated a dramatic improvement in LV function and trace paravalvular leak.

## Discussion

There are limited mechanical circulatory support (MCS) options in patients presenting with cardiogenic shock in the setting of biventricular failure and severe aortic regurgitation. VA-ECMO is contraindicated in this patient population because the increased afterload from the VA-ECMO cannulation would result in LV dilatation, severe pulmonary edema, and the risk of thrombus formation in the LV. In fact, catastrophic effects on LV function have been reported in cases with even mild aortic regurgitation.^1^ The use of 2 univentricular devices, such as Impella (Abiomed Inc) or Tandem Heart (LivaNova), can be considered; however, Impella is also contraindicated in patients with severe AI, and the use of 2 devices can increase the risk of complications and hemolysis, particularly in patients who will require MCS support for longer than a few days. The use of surgically implanted VA-ECMO with the inflow cannula directly placed in the LV or the use of a biventricular ventricular assist device can also provide adequate support but comes with the prerequisite of surgical implantation (which is considered a prohibitive risk in patients who are not being considered for a durable LV assist device or transplantation). Hence, LAVA-ECMO serves as a viable percutaneous option for such patients.

There is an ongoing increase in the global awareness of and demand for early implementation of MCS in cardiogenic shock patients, which has been associated with improved survival. However, there remain limitations to existing MCS technologies. Some forms of MCS are not readily available in an emergency setting (eg, a biventricular ventricular assist device), some are contraindicated in certain clinical scenarios (eg, VA-ECMO in severe aortic regurgitation), and others remain expensive or require multiple large-bore accesses (eg, VA-ECMO plus Impella). LAVA-ECMO was first described in 2018 for patients with biventricular cardiogenic shock.^2^ In our case, we demonstrated its effectiveness even in cardiogenic shock patients with severe aortic regurgitation with improvement of invasive hemodynamics before and after cannulation. The placement of LAVA-ECMO is not technically challenging in centers performing transseptal procedures regularly and is also more economical and available worldwide. In conclusion, LAVA-ECMO can be used as an MCS strategy for patients with cardiogenic shock with severe aortic insufficiency.

## Follow-Up

Transthoracic echocardiogram performed 1 day after TAVR demonstrated normal LV ejection fraction (63%). The patient was discharged from the hospital 3 days after TAVR.

## Conclusions

LAVA-ECMO is an effective, non–technically demanding, and cost-effective option for hemodynamic support in patients with cardiogenic shock and severe aortic regurgitation.

## Funding Support and Author Disclosures

Dr B.P. O’Neill is a consultant to and receives research support from Edwards Lifesciences. Dr Frisoli is a proctor for Edwards Lifesciences, Abbott, Boston Scientific, and Medtronic. Dr Wang is a consultant for Edwards Lifesciences, Abbott, Neochord, and Boston Scientific and receives research grant support from Boston Scientific assigned to employer Henry Ford Health System. Dr W.W. O’Neill has served as a consultant for Abiomed, Edwards Lifesciences, Medtronic, Boston Scientific, Abbott Vascular, and St. Jude Medical and serves on the Board of Directors of Neovasc Inc. Dr Villablanca is a consultant for Edwards Lifesciences and Teleflex. All other authors have reported that they have no relationships relevant to the contents of this paper to disclose.

## Appendix

Supplemental Video 1 Aortogram demonstrating severe aortic regurgitation, which was immediately drained out of the LV into the LA and then into the LAVA-ECMO cannula, decompressing the left heart.

Supplemental Video 2 Alternating oxygenated (brighter from the LA) and deoxygenated (darker from the RA) blood in the venous LAVA-ECMO cannula.

Supplemental Video 3A (A) Final angiogram after percutaneous coronary intervention of left coronary arteries. (B) Final aortogram after TAVR with left main protection.

Supplemental Video 3B (A) Final angiogram after percutaneous coronary intervention of left coronary arteries. (B) Final aortogram after TAVR with left main protection.4
